# Respiratory syncytial virus epidemiology and effectiveness of infant nirsevimab: 2024 results from the Australian Sentinel Hospital Network (FluCAN-PAEDS)

**DOI:** 10.2807/1560-7917.ES.2026.31.2.2500275

**Published:** 2026-01-15

**Authors:** Christopher C Blyth, Ushma Wadia, Philip N Britton, Jeremy Carr, Julia E Clark, Nigel W Crawford, Te-Yu Hung, Kristine K Macartney, Helen S Marshall, Nicholas J Wood, Tom Kotsimbos, Paul M Kelly, Allen C Cheng, FluCAN-PAEDS network, FluCAN-PAEDS network, Mark Holmes, Louis Irving, Stephen Vincent, Sanjaya Senanayake, Tony Korman, Caroline Bartolo, Louise Cooley, Peter Wark, Simon Bowler, Jen Kok, Naomi Runnegar, Daniel Fatovich, Grant Waterer, Anne Kynaston, Brendan McMullan, Joshua Francis, Peter Richmond

**Affiliations:** 1 University of Western Australia/The Kids Research Institute Australia, Perth, Australia; 2 Perth Children's Hospital, Perth, Australia; 3 Pathwest Laboratory Medicine, Perth, Australia; 4 The Children's Hospital at Westmead, Sydney, Australia; 5 Faculty of Medicine and Health, University of Sydney, Sydney, Australia; 6 National Centre of Immunisation Research & Surveillance, Sydney, Australia; 7 Monash Children's Hospital, Melbourne, Australia; 8 Monash University, Melbourne, Australia; 9 Queensland Children's Hospital, Brisbane, Australia; 10 Royal Children's Hospital, Melbourne, Australia; 11 Murdoch Children's Research Institute and Department Paediatrics, University of Melbourne, Australia; 12 Royal Darwin Hospital, Darwin, Australia; 13 Menzies School of Health Research, Darwin, Australia; 14 Women's and Children's Hospital, Adelaide, Australia; 15 University of Adelaide, Adelaide, Australia; 16 Alfred Hospital, Melbourne, Australia; 17 Commonwealth Department of Health, Disability and Ageing, Canberra, Australia; 18 Australian National University, Australian Capital Territory, Australia; 19 Monash Health, Melbourne, Australia; 20 Additional members of the FluCAN-PAEDS network are listed under Collaborators and at the end of the article.

**Keywords:** respiratory syncytial virus, acute respiratory infection, immunisation, nirsevimab, Australia, vaccine effectiveness

## Abstract

**BACKGROUND:**

Respiratory syncytial virus (RSV) is a leading cause of morbidity and mortality in young children and older adults. A long-acting anti-RSV monoclonal antibody (nirsevimab) and bivalent pre-fusion F-protein pregnancy vaccine became available to prevent RSV in young children in 2024; two RSV vaccines for adults ≥ 60 years were also available.

**AIM:**

To report 2024 RSV epidemiology in Australia, identify risk factors for severe outcomes, and use and effectiveness of RSV immunisation products.

**METHODS:**

National sentinel hospital-based RSV surveillance was established in 2024, recruiting hospitalised laboratory-confirmed RSV cases and test-negative controls from 22 sites in a national hospital network (FluCAN-PAEDS).

**RESULTS:**

Between April and December 2024, 3,998 subjects (3,415 children; 582 adults) were hospitalised with RSV. Most cases were infants < 12 months (n = 1,534; 38.4%); 1,661 (41.5%) had underlying medical conditions. Children < 6 months, First Nations children, those born preterm or with underlying medical conditions (cardiac, neurological, genetic and metabolic disease/disorders, immunosuppression) were at greatest risk of severe outcomes. Severe outcomes were more frequent in adults with malignancy, respiratory or cardiac disease. Nirsevimab effectiveness against hospitalisation in infants < 12 months in the two Australian jurisdictions with population-wide immunisation programmes was 83.1% (95% CI: 67.4–91.3). RSV vaccine use (pregnancy; adults ≥ 60 years) was limited, precluding effectiveness assessments.

**CONCLUSION:**

National surveillance enabled timely 2024 data collection with the capability to evaluate effectiveness of immunisation products preventing RSV. Nirsevimab demonstrated comparable effectiveness to that in the northern hemisphere, informing Australia’s 2025 strategy. Evaluation to assess the impact of more widespread uptake of RSV prevention products continues.

**Key public health message**

**What did you want to address in this study and why?**

Respiratory syncytial virus (RSV) can cause acute respiratory tract infection. We sought to establish national hospital-based RSV surveillance to explore demographics, risk factors and outcomes for RSV-associated hospitalisation in children and adults, and assess the effectiveness of immunisation products preventing RSV available in Australia in 2024 to inform current and future RSV prevention programmes.

**What have we learnt from this study?**

Despite increased RSV testing in adults, young children remain most likely to be hospitalised with RSV. One in 16 children and one in 10 adults hospitalised with RSV are admitted to the ICU. In-hospital mortality is rare in children (0.1%) but was observed in approximately 1 in 25 hospitalised adults. Immunisation effectiveness of nirsevimab against hospitalisation in infants < 12 months in two Australian jurisdictions was over 80%.

**What are the implications of your findings for public health?**

Given the demonstrated effectiveness of nirsevimab and 2025 expansion of the Australian infant RSV prevention programme to include a maternal vaccine, promotion and ongoing evaluation is required to ensure impacts are observed Australia-wide. The impact of RSV in older or medically-at-risk adult populations is notable, given that RSV vaccines are now available to prevent morbidity and mortality.

## Introduction

Globally, respiratory syncytial virus (RSV) is a leading cause of acute respiratory tract infection, with young children and older adults aged ≥ 60 years at particular risk of morbidity and mortality [1,2]. Most children are infected with RSV in the first 2 years of life with reinfections occurring throughout an individual’s lifetime. In Australia, those at greatest risk of RSV-associated complications, including hospitalisation, are young infants (< 6 months), those born preterm, First Nations children and children with underlying medical conditions [3-5]. Older individuals (≥ 60 years), particularly those with underlying cardiorespiratory disease, are also at increased of RSV-associated complications [2,6,7]. Data on RSV-associated deaths in adults are less robust; in young children, deaths are rare in high-income countries like Australia, with 97% of RSV infant mortality occurring in low- and middle-income countries [8].

The COVID-19 pandemic and associated public health and social measures resulted in notable disruption to the expected RSV seasonality, both globally and in Australia [9,10]. Since 2023, RSV epidemiology in Australia has returned to the traditional winter peaks, observed in the temperate southern jurisdictions, with year-round activity observed in the tropical north [11].

Clinical trials demonstrating the safety and efficacy of recombinant long-acting anti-RSV monoclonal antibodies against RSV disease in young children (nirsevimab, Beyfortus, Sanofi-Aventis Australia, November 2023; clesrovimab, Enflonsia, MSD, not yet registered in Australia) [12,13] and vaccination in pregnancy using a recombinant RSV pre-fusion F-protein bivalent vaccine [14] (Abrysvo, Pfizer Australia, March 2024) have led to approval of these products both internationally and in Australia. Similarly, safety and efficacy of the adjuvanted recombinant RSV pre-fusion F-protein vaccine [15] (RSVpreF3 OA, Arexvy, GlaxoSmithKline Australia, January 2024) and RSVpreF [16] in adults underpinned approval for RSV prevention in older populations.

Use of RSV immunisation products in Australia in 2024 varied across the six states and two territories (referred to collectively as jurisdictions). Individual jurisdictions established nirsevimab programmes for young children early in 2024, ranging from population-wide approaches in Western Australia (WA) and Queensland, to very targeted risk-based programmes in other jurisdictions. 

Given availability and use of RSV prevention products in Australia, we sought to establish real-time national hospital-based RSV surveillance in all age groups. Utilising an established national hospital network, FluCAN-PAEDS, which conducts influenza and SARS-CoV-2 surveillance and operates in all Australian jurisdictions, we report on the epidemiology of RSV in Australia in 2024, risk factors for severe outcomes, use and effectiveness (where possible) of available RSV prevention products.

## Methods

### Study setting

**Figure 1:**
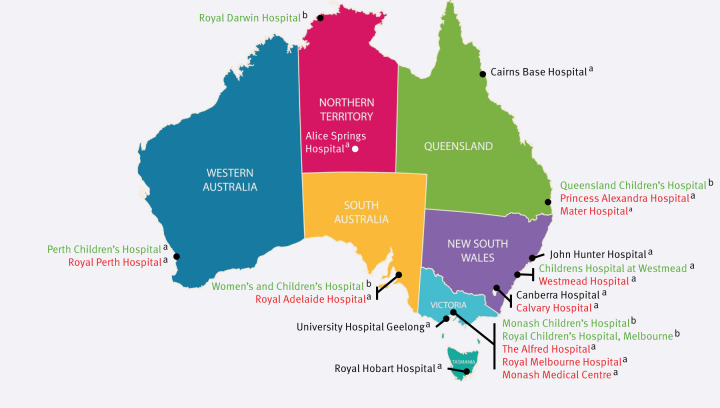
FluCAN-PAEDS sentinel hospitals participating in RSV surveillance, Australia, 2024–2025 (n = 22 hospitals)

FluCAN: Influenza Complications Alert Network; PAEDS: Paediatric Active Enhanced Disease Surveillance; RSV: respiratory syncytial virus.

^a^ Hospitals collecting data since 2011 (n = 17).

^b^ Hospitals collecting data since 2017 (n = 5).

The Influenza Complications Alert Network (FluCAN) is a national hospital-based sentinel surveillance system with the capacity to activate data collection for new infections or infections of specific public health interest [17]. Seventeen hospitals have contributed data since 2011 (Figure 1) with five additional specialist paediatric hospitals belonging to the Paediatric Active Enhanced Disease Surveillance (PAEDS) programme contributing data since 2017 [18].

Hospitals are located in every state (New South Wales, Victoria, Queensland, Western Australia, South Australia and Tasmania) and territories (Northern Territory, Australia Capital Territory (Canberra and Calvary hospitals)). Hospital names are colour coded as green: paediatric recruitment only, red: adult recruitment only, and black: both paediatric and adult recruitment.

Data have been collected through FluCAN-PAEDS for cases of hospitalised influenza since establishment in 2009 and hospitalised COVID-19 in all sentinel hospitals since 2020. In April 2024, surveillance for hospitalised episodes of RSV commenced at all FluCAN and PAEDS sites funded by the Australian Commonwealth Department of Health [11]. Given RSV is highly seasonal in temperate regions of Australia (May–September) but more variable and distributed across the year in warmer predominantly northern regions, year-round data collection was implemented. 

### RSV immunisation programmes in 2024

In 2024, WA and Queensland launched population-wide RSV prevention programmes for infants and young children utilising nirsevimab. From 2 April 2024, WA recommended nirsevimab to: (i) all infants born from 1 October 2023 to 30 September 2024; (ii) a selection of medically at-risk children born after 30 September 2022 and (iii) First Nations children born after 30 September 2022, given their increased risk of severe disease and access barriers to health care. Given year-round RSV activity, the WA programme was extended for children living in the tropical north of the state born after the 30 September 2024. From 15 April 2024, Queensland commenced a year-round programme, recommending nirsevimab to: (i) all infants born or younger than 8 months of age at the date of programme commencement and (ii) medically at-risk children aged 8 months to 19 months inclusive. Other jurisdictions restricted use of nirsevimab to young infants with additional risk factors, e.g. preterm birth, First Nations background and medically at-risk children, putting them at highest risk for severe RSV disease.

RSVpreF for pregnant people and RSVpreF3 OA for older individuals were available for private purchase in 2024 but not funded by the National Immunisation Program (NIP). Queensland made Abrysvo available to all pregnant individuals from 1 December 2024. Other jurisdictions did not include RSV vaccines in state-based programmes.

### Identification of cases and controls

RSV surveillance was undertaken between 1 April and 31 December 2024. A case was defined as a patient with respiratory symptoms admitted to the hospital with RSV, confirmed by nucleic acid testing (NAT). In sentinel hospitals, testing is routinely performed on patients with acute respiratory infection (ARI) to aid management and infection control practices, using validated NAT assays. Data from a sample of patients who were admitted with an ARI but tested negative for RSV were collected as controls. To ensure sufficient and appropriate age distribution, a set number of controls, defined a priori, were identified from hospitals admitting paediatric and adult cases (number of controls per week: two children aged < 12 months, two children aged 1 to < 5 years and one child aged 5 to < 17 years from hospitals admitting paediatric cases; one adult aged 18 to < 40 years, two adults aged 40–59 years and two adults aged ≥ 60 years from hospitals admitting adult cases).

### Data collection

The onset date was defined as the date of admission. For patients where the date of the test was more than 7 days after admission, the onset date was the date of the test. Infections occurring more than 7 days after admission were considered nosocomial infections. Admission or transfer to an intensive care unit (ICU) included patients managed in a designated high dependency unit (HDU). The presence of risk factors, preterm birth (< 37 weeks gestation) and medical comorbidities (underlying cardiac disease, respiratory disease, renal disease, neurological disease, liver disease, malignancy, immunosuppression, genetic disorder, metabolic disorder, diabetes mellitus and obesity) were ascertained from the patient’s medical record. First Nations status included all Aboriginal Australian and/or Torres Strait Islander people based on self-report on medical records. Prior immunisation receipt (including receipt of pregnancy vaccine for infants < 6 months) was determined from the AIR.

### Statistical analyses

Demographics, risk factors, underlying medical conditions and outcomes were explored using descriptive statistics. Categorical data were described using proportions and associations explored using chi-square tests. Continuous data were described using median values and interquartile ranges and associations explored using Mann–Whitney tests. We examined factors associated with ICU admission using multivariable regression. Factors independently associated with ICU admission were determined using a logistic regression model. We modelled factors associated with length of hospital stay using negative binomial regression, where the exponential of the regression coefficient represents the relative increase in hospital length of stay. In addition to age, sex and First Nations status, predictors with a p < 0.1 in the univariate analyses were retained and multivariable models fitted using the Akaike Information Criterion (AIC).

Use of RSV immunisation products (infant nirsevimab, RSVpreF in pregnancy, RSVpreF3 OA in older individuals) was estimated from the proportion of immunised individuals in test-negative controls in eligible age groups. Immunisation effectiveness (IE) was estimated from the odds ratio (OR) of immunisation in cases vs controls using the formula IE (%) = (1−adjusted odds ratio (aOR)) x 100, with the odds ratio calculated from a conditional logistic regression, stratified by jurisdiction and week, and adjusted for age group, the presence of underlying medical conditions and preterm birth, and First Nations status. A priori power calculations estimated that 182 cases and 182 controls were required to reject the null hypothesis that there was no effect of immunisation on hospitalisation with 90% power at the p < 0.05 level of significance, if 50% of controls were immunised.

Analysis was undertaken using STATA v18.0.

## Results

**Figure 2:**
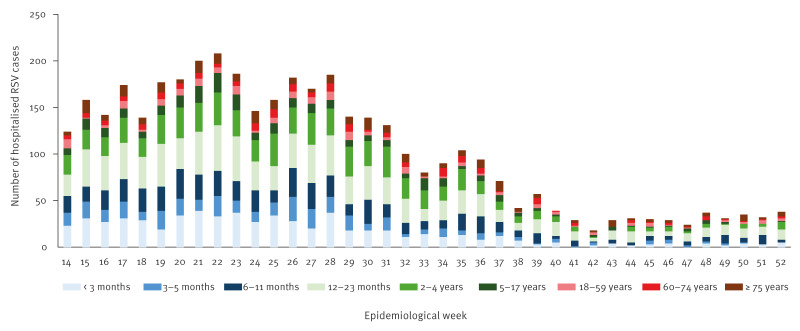
Number of RSV cases per week by age group, Australia, 2024 (n = 3,998)

A total of 3,998 RSV cases were recruited into the existing FluCAN-PAEDS surveillance programme between 1 April and 31 December 2024, including 3,416 paediatric (age < 18 years) and 582 adult cases (Figure 2). Early RSV activity was identified in New South Wales (peak: week 17), Queensland (peak: week 17) and Victoria (peak: week 22) with later activity noted in South Australia (peak: week 28) and Western Australia (peak: week 31). The number of RSV cases per week by jurisdiction is provided in Supplementary Figure S1.

RSV: respiratory syncytial virus.

As cases were recruited from 1 April 2024, weeks 1 to 13 are not displayed. Data source: FluCAN-PAEDS Surveillance data.

### Demographics and risk factors

Of all cases, 1,534 (38.4%) were infants aged < 12 months (Table 1). A total of 268 RSV cases (6.7%) were identified as being of First Nations background (paediatric: 6.0%; adult 10.8%; p < 0.001) and 1,661 (41.5%) were reported to have underlying medical conditions (paediatric: 33.9%; adult: 86.6%; p < 0.001). Seventy-eight episodes were considered nosocomial (paediatric: n = 36, 1.1%; adult: n = 42, 7.2%).

**Table 1:** Demographic, risk factors and outcomes for hospitalised RSV cases by jurisdiction (n = 3,998) and test-negative controls (n = 3,425), Australia, 2024

Characteristics	RSV cases by jurisdiction																Total RSV cases (n = 3,998)			Test-negative controls (n = 3,425)	
	New South Wales (n = 566)		Victoria (n = 1,510)		Queensland (n = 452)		Western Australia (n = 647)		South Australia (n = 552)		Tasmania (n = 80)		Northern Territory (n = 161)		Australia Capital Territory (n = 30)^a^						
	n	%	n	%	n	%	n	%	n	%	n	%	n	%	n	%	n		%	n	%
**Age group**																					
< 3 months	102	18.0	293	19.4	41	9.1	46	7.1	116	21.0	9	11.3	17	10.6	0	0	624		15.6	218	6.4
3–5 months	71	12.5	146	9.7	25	5.5	27	4.2	58	10.5	7	8.8	10	6.2	0	0	344		8.6	97	2.8
6–11 months	94	16.6	211	14.0	64	14.2	69	10.7	93	16.8	10	12.5	25	15.5	0	0	566		14.2	205	6.0
12–23 months	134	23.7	305	20.2	123	27.2	205	31.7	127	23.0	13	16.3	29	18.0	0	0	936		23.4	250	7.3
2–4 years	71	12.5	233	15.4	86	19.0	187	28.9	72	13.0	7	8.8	23	14.3	0	0	679		17.0	293	8.5
5–17 years	40	7.1	112	7.4	27	6.0	36	5.6	34	6.2	3	3.8	15	9.3	0	0	267		6.7	296	8.6
18–59 years	15	2.7	62	4.1	21	4.6	19	2.9	12	2.2	8	10.0	20	12.4	5	16.7	162		4.1	1,066	31.1
60–74 years	14	2.5	60	4.0	20	4.4	22	3.4	17	3.1	5	6.3	15	9.3	11	36.7	164		4.1	445	13.0
≥ 75 years	25	4.4	88	5.8	45	10.0	36	5.6	23	4.2	18	22.5	7	4.3	14	46.7	256		6.4	555	16.2
**Sex**																					
Male	312	55.1	792	52.5	238	52.7	347	53.6	298	54.0	36	45.0	83	51.6	14	46.7	2120		53.0	1,818	53.1
Female	243	42.9	716	47.5	214	47.3	300	46.4	254	46.0	44	55.0	78	48.4	16	53.3	1865		46.6	1,604	46.8
Other/not reported	11	1.9	2	0.1	0	0	0	0	0	0	0	0	0	0	0	0	13		0.3	3	<0.1
**Ethnicity^b^**																					
First Nations	14	2.5	36	2.4	37	8.2	57	8.8	27	4.9	12	15.0	82	50.9	3	10.0	268		6.7	383	11.2
Not First Nations	552	97.5	1,474	97.6	415	91.8	590	91.2	525	95.1	68	85.0	79	49.1	27	90.0	3,730		93.3	3,042	88.8
**Underlying medical conditions**																					
Yes	224	39.6	657	43.5	255	56.4	187	28.9	198	35.9	39	48.8	72	44.7	29	96.7	1,661	41.5		2,165	63.2
No	342	60.4	853	56.5	197	43.6	460	71.1	354	64.1	41	51.3	89	55.3	1	3.3	2,337	58.5		1,260	36.8
**Admitted to ICU or HDU**																					
Yes	43	7.6	109	7.2	38	8.4	33	5.1	23	4.2	4	5.0	9	5.6	6	20.0	265	6.6		296	8.6
No	523	92.4	1401	92.8	414	91.6	614	94.9	529	95.8	76	95.0	152	94.4	24	80.0	3,733	93.4		3,129	91.4
**In-hospital mortality**																					
Yes	1	0.2	11	0.7	2	0.4	6	0.9	5	0.9	1	1.3	1	0.6	1	3.3	28	0.7		60	1.8
No	565	99.8	1499	99.3	450	99.6	641	99.1	547	99.1	79	98.8	160	99.4	29	96.7	3,970	99.3		3,365	98.2

HDU: high dependency unit; ICU: intensive care unit; RSV: respiratory syncytial virus.

^a^ No paediatric cases recruited from the Australian Capital Territory.

^b^ First Nations status included all Aboriginal Australian and Torres Strait Islander people based on self-report on medical records.

### Outcomes

Admission to ICU/HDU was required in 265 RSV cases (6.6% overall; n = 210 (6.1%) in children vs n = 55 (9.5%) in adults; p < 0.01). Twenty-eight in-hospital deaths were observed (0.7% overall; n = 4 (0.1%) in children and n = 24 (4.1%) in adults; p < 0.001). Fourteen deaths (50%) occurred in those ≥ 75 years and all but one death (a child) occurred in individuals with underlying medical conditions. The median length of stay was shorter in children (2 days (IQR: 1–3) vs 4 (IQR: 3–9) in adults; p < 0.001) as was the proportion with a hospital length of stay > 7 days (12.7% vs 41.1%; p < 0.001).

Risk factors for ICU/HDU admission and length of stay > 7 days were explored separately for children and adults. Children aged < 6 months and those with underlying medical conditions were overrepresented in those requiring ICU/HDU admission and with a prolonged length of stay (Table 2). In multivariable models, children aged < 6 months, those born preterm, children with cardiac or neurological disease, or genetic or metabolic disorders were more likely admitted to ICU/HDU (Table 3) whereas those children aged < 6 months, First Nations children, those with underlying medical conditions, especially cardiac and neurological disease as well as malignancy and immunosuppression were more likely to have a prolonged length of stay (Table 3).

**Table 2:** Risk factors associated with severe RSV disease in children (< 18 years) and adults, Australia, 2024 (n = 3,998)

Characteristics	ICU/HDU admission				Length of stay > 7 days				Total	
	n	%	n	%	n	%	n	%	n	%
Children	Yes (n = 210)		No (n = 3,206)		Yes (n = 433)		No (n = 2,983)		(n = 3,416)	
**Age group**										
< 3 months	64	30.5	560	17.5	114	26.3	510	17.1	624	18.3
3–5 months	21	10.0	323	10.1	61	14.1	283	9.5	344	10.1
6–11 months	27	12.9	539	16.8	75	17.3	491	16.5	566	16.6
12–23 months	36	17.1	900	28.1	74	17.1	862	28.9	936	27.4
2–4 years	34	16.2	645	20.1	61	14.1	618	20.7	679	19.9
5–17 years	28	13.3	239	7.5	48	11.1	219	7.3	267	7.8
**Sex**										
Male	110	52.4	1,754	54.7	225	52.0	1,639	54.9	1,864	54.6
Female	100	47.6	1,439	44.9	196	45.3	1,343	45.0	1,539	45.0
Other/not reported	0	0	13	0.4	12	2.8	1	< 0.1	13	0.4
**Ethnicity^a^**										
First Nations	18	8.6	187	5.8	30	6.9	175	5.9	205	6.0
Not First Nations	192	91.4	3,019	94.2	403	93.1	2,808	94.1	3,211	94.0
**Underlying medical conditions^b^**										
Yes	116	55.2	1,041	32.5	199	46.0	958	32.1	1,157	33.9
Preterm birth	55	26.2	348	10.9	53	12.2	350	11.7	403	11.8
Cardiac disease	31	14.8	131	4.1	42	9.7	120	4.0	162	4.7
Respiratory disease	49	23.3	368	11.5	66	15.2	351	11.8	417	12.2
Renal disease	7	3.3	50	1.6	11	2.5	46	1.5	57	1.7
Neurological disease	30	14.3	116	3.6	41	9.5	105	3.5	146	4.3
Liver disease	2	1.0	13	0.4	3	0.7	12	0.4	15	0.4
Malignancy	1	0.5	74	2.3	17	3.9	58	1.9	75	2.2
Immunosuppression	4	1.9	87	2.7	18	4.2	73	2.4	91	2.7
Genetic disorder	30	14.3	80	2.5	26	6.0	84	2.8	110	3.2
Metabolic disorder^c^	4	1.9	8	0.2	4	0.9	8	0.3	12	0.4
Diabetes mellitus	1	0.5	8	0.2	2	0.5	7	0.2	9	0.3
No	94	44.8	2,165	67.5	234	54.0	2,025	67.9	2,259	66.1
Adults	Yes (n = 55)		No (n = 527)		Yes (n = 239)		No (n = 343)		(n = 582)	
**Age group**										
18–59 years	25	45.5	137	26.0	43	18.0	119	34.7	162	27.8
60–74 years	17	30.9	147	27.9	76	31.8	88	25.7	164	28.2
≥ 75 years	13	23.6	243	46.1	120	50.2	136	39.7	256	44.0
**Sex**										
Male	28	50.9	228	43.3	101	42.3	155	45.2	256	44.0
Female	27	49.1	299	56.7	138	57.7	188	54.8	326	56.0
**Ethnicity^a^**										
First Nations	10	18.2	53	10.1	15	6.3	48	14.0	63	10.8
Not First Nations	45	81.8	474	89.9	224	93.7	295	86.0	519	89.2
**Underlying medical conditions^b^**										
Yes	48	87.3	456	86.5	215	90.0	289	84.3	504	86.6
Cardiac disease	20	36.4	198	37.6	95	39.7	123	35.9	218	37.5
Respiratory disease	36	65.5	196	37.2	94	39.3	138	40.2	232	39.9
Renal disease	7	12.7	76	14.4	29	12.1	54	15.7	83	14.3
Neurological disease	6	10.9	86	16.3	44	18.4	48	14.0	92	15.8
Liver disease	2	3.6	16	3.0	8	3.3	10	2.9	18	3.1
Malignancy	9	16.4	100	19.0	61	25.5	48	14.0	109	18.7
Immunosuppression	15	27.3	124	23.5	65	27.2	74	21.6	139	23.9
Diabetes mellitus	30	54.5	280	53.1	126	52.7	184	53.6	310	53.3
Obesity	5	9.1	57	10.8	19	7.9	43	12.5	62	10.7
No	7	12.7	71	13.5	24	10.0	54	15.7	78	13.4
**Other risk factors**										
Pregnancy	1	1.8	7	1.3	2	0.8	6	1.7	8	1.4
Current smoker	13	23.6	75	14.2	33	13.8	55	16.0	88	15.1
Nursing home residence	0	0.0	45	8.5	13	5.4	32	9.3	45	7.8

HDU: high dependency unit; ICU: intensive care unit; RSV: respiratory syncytial virus.

^a^ First Nations status included all Aboriginal Australian and Torres Strait Islander people based on self-report on medical records.

^b^ Multiple medical conditions reported per patient.

^c^ Excluding diabetes mellitus.

**Table 3:** Predictors of intensive care/high dependency unit admission and prolonged length of stay in children (< 18 years) and adults with RSV disease, Australia, 2024 (n = 3,998)

Variables	Crude OR	95% CI	p value	Adjusted OR^a^		95% CI		p value		
**Predictors of ICU and HDU admission**										
**Children**										
Age < 6 months	1.79	1.34–2.38	< 0.001		2.71	1.97–3.73				< 0.001
Male sex	1.11	0.84–1.47	0.472		NS					
First Nations status^b^	1.51	0.91–2.50	0.108		NS					
Any underlying medical condition	2.56	1.94–3.40	< 0.001		NS					
Preterm birth	2.91	2.10–4.04	< 0.001		2.03		1.34–3.07		0.001	
Cardiac disease	4.06	2.67–6.18	< 0.001		1.83		1.13–2.98		0.014	
Respiratory disease	2.34	1.67–3.29	< 0.001		NS					
Neurological disease	4.44	2.89–6.81	< 0.001		2.27		1.37–3.77		0.001	
Genetic disorder	6.51	4.17–10.17	< 0.001		3.69		2.17–6.27		< 0.001	
Metabolic disorder excluding diabetes	7.76	2.32–26.00	0.001		4.55		1.16–17.82		0.030	
**Adults**										
Age ≥ 75 years	0.36	0.19–0.69	0.002		0.40		0.21–0.78		0.007	
Male sex	0.74	0.42–1.29	0.278		NS					
First Nations status^b^	1.98	0.94–4.16	0.070		NS					
Respiratory disease	3.20	1.79–5.73	< 0.001		3.16		1.75–5.70		< 0.001	
**Predictors of prolonged length of stay**										
**Children**										
Age < 6 months	1.26	1.07–1.49	0.006		1.54		1.36–1.73	< 0.001		
Male sex	0.96	0.88–1.05	0.346		NS					
First Nations status^b^	1.17	0.97–1.40	0.096		1.21		1.04–1.42	0.016		
Any underlying medical condition	1.92	1.51–2.43	< 0.001		1.40		1.27–1.54	< 0.001		
Preterm birth	1.58	1.17–2.15	0.003		NS					
Cardiac disease	2.31	1.86–2.88	< 0.001		1.57		1.31–1.87		< 0.001	
Respiratory disease	1.49	1.22–1.82	< 0.001		NS					
Neurological disease	2.42	1.88–3.11	< 0.001		1.96		1.48–2.60		< 0.001	
Malignancy	2.99	2.01–4.44	< 0.001		1.94		1.44–2.59		< 0.001	
Immunosuppression	2.75	2.05–3.69	< 0.001		1.49		1.08–2.07		0.016	
Genetic disorder	2.08	1.54–2.79	< 0.001		NS					
Metabolic disorder excluding diabetes	2.35	1.21–4.58	0.012		NS					
**Adults**										
Age ≥ 75 years	1.28	1.00–1.64	< 0.001		NS					
Male sex	1.06	0.92–1.22	0.408		NS					
First Nations status^b^	0.66	0.51–0.86	0.002		NS					
Any underlying medical condition	1.27	0.99–1.64	0.063		NS					
Cardiac disease	1.19	0.99–1.43	0.062		1.15		1.00–1.31		0.038	
Malignancy	1.38	0.06–1.79	0.018		1.28		1.00–1.65		0.048	
Immunosuppression	1.19	1.00–1.43	0.049		NS					
Diabetes mellitus	0.83	0.70– 0.99	0.034		0.83		0.72–0.96		0.011	

CI: confidence interval; HCU: high dependency unit; ICU: intensive care unit; OR: odds ratio; RSV: respiratory syncytial virus.

^a^ In addition to age, sex and First Nations status, factors associated with p value < 0.1 in univariate models were included in the multivariable model.

^b^ First Nations status included all Aboriginal Australian and Torres Strait Islander people based on self-report on medical records.

Children < 18 years and adults ≤ 18 years. Reference variables include all others in the cohort.

Younger and First Nations adults were overrepresented in those requiring ICU/HDU admission whereas older adults were overrepresented in those with prolonged length of stay (Table 2B). In multivariable models, adults aged 18–74 years and those with respiratory conditions were more likely admitted to ICU/HDU (Table 3). Adults with underlying cardiac disease or malignancy were more likely to have a prolonged length of stay (Table 3) and those with diabetes more likely to have a shorter length of stay.

### RSV prevention product use and effectiveness

Use of RSV prevention products varied significantly by jurisdiction (p < 0.001). Overall, 73 of 442 (16.5%) of RSV test-negative infants < 12 months received nirsevimab. However, 37 of 77 (48.1%) and 21 of 80 (26.2%) of RSV negative controls aged < 12 months in WA and Queensland, respectively, received nirsevimab compared with 15 of 285 (5.3%) in other jurisdictions (Table 4). The impact of this was evident in the proportion of total paediatric RSV cases detected in children aged < 12 months which was significantly lower in WA (24.9%) and Queensland (35.5%) compared with other jurisdictions combined (50.9%; p < 0.001; the number of paediatric cases by age group and jurisdiction is provided in Supplementary Figure S2). Nirsevimab use was reported in only 10 of 221 (4.5%) RSV test-negative children aged 12–23 months (n = 4/31 in WA; n = 3/36 in Queensland and n = 3/154 in other jurisdictions). No use of RSVpreF vaccine in pregnancy was reported for infant cases or controls. Receipt of RSVpreF3 OA vaccine was reported in only eight adults aged 60 years and older (three RSV cases; five RSV negative controls).

**Table 4:** Use of RSV prevention products by age group in jurisdictions with (Queensland and Western Australia) and without population-based nirsevimab programmes, Australia, 2024

Age and case-control status		Jurisdiction																																				
		New South Wales				Victoria				Queensland^a^				Western Australia^a^				South Australia				Tasmania				Northern Territory				Australian Capital Territory				Total				
		Imm		Unimm		Imm		Unimm		Imm		Unimm		Imm		Unimm		Imm		Unimm		Imm		Unimm		Imm		Unimm		Imm		Unimm		Imm		Unimm	
Age	Group	n	%	n	%	n	%	n	%	n	%	n	%	n	%	n	%	n	%	n	%	n	%	n	%	n	%	n	%	n	%	n	%	n	%	n	%
0–11 months	Cases	1	0.5	203	99.5	0	0	488	100	10	8.0	115	92.0	27	20.2	107	79.8	2	1.1	187	98.9	0	0	18	100	0	0	50	100	0	0	0	0	40	3.3	1,168	96.7
	Controls	2	9.1	20	90.9	8	5.8	131	94.2	21	26.2	59	73.8	37	48.1	40	51.9	2	2.5	77	97.5	0	0	0	0	3	6.7	42	93.3	0	0	0	0	73	16.5	369	83.5
12–23 months	Cases	0	0	108	100	1	0.4	255	99.6	5	4.3	112	95.7	7	3.5	193	96.5	0	0	99	100	0	0	11	100	0	0	29	100	0	0	0	0	13	1.6	807	98.4
	Controls	1	7.7	12	92.3	2	2.7	72	97.3	3	8.3	33	91.7	4	12.9	27	87.1	0	0	33	100	0	0	0	0	0	0	34	100	0	0	0	0	10	4.5	211	95.5
≥ 60 years	Cases	0	0	0	0	2	5.3	36	94.7	0	0	52	100	0	0	56	100	1	3.0	32	97.0	0	0	20	100	0	0	3	100	0	0	22	100	3	1.3	221	98.7
	Controls	1	2.8	35	97.2	3	1.9	154	98.1	1	0.4	226	99.6	0	0	74	100	0	0	40	100	0	0	20	100	0	0	20	100	0	0	53	100	5	0.8	622	99.2

Imm: immunised; RSV: respiratory syncytial virus; Unimm: unimmunised.

^a^ Jurisdictions with population-based nirsevimab programmes are indicated in grey.

Cases and controls aged 0–11 months in Queensland and WA: n = 416 versus other jurisdictions: n = 1,234. Cases and controls aged 12–23 months in Queensland and WA: n = 384 versus other jurisdictions: n = 657. Cases and controls aged ≥ 60 in all jurisdictions: n = 851.

No mothers of infant cases or controls were reported to have received the maternal RSVpreF vaccine.

Immunisation effectiveness (IE) against hospitalisation was estimated for nirsevimab in the two jurisdictions with population-wide programmes (WA and Queensland; combined birth cohort of approximately 90,000 births) (Table 5). The IE was estimated to be 83.1% (95% confidence interval: 67.4 to 91.3) in infants aged < 12 months. Nirsevimab use in the second year of life was insufficient to estimate its effectiveness. A trend towards effectiveness against RSV-associated ICU/HDU admission was observed (91.5%; 95% CI: −61.1 to 99.5); however, small numbers preclude further conclusions on effectiveness against severe outcomes. Exclusion of nosocomial cases had no impact on immunisation effectiveness estimates.

**Table 5:** Immunisation effectiveness against RSV hospitalisation in children (< 2 years) residing in jurisdictions with population-based nirsevimab programmes, Queensland and Western Australia, Australia, 2024 (n = 800)

Immunisation effectiveness by age	Number of RSV-positive cases		Number of RSV-negative controls		OR	95% CI	aOR	95% CI	Nirsevimab effectiveness	95% CI
	Imm	Unimm	Imm	Unimm						
**RSV hospitalisation**										
< 6 months	26	108	34	56	0.397	0.217 to 0.725	0.191^a^	0.071 to 0.513	80.9%^a^	48.7 to 92.9
< 12 months	37	222	58	99	0.284	0.177 to 0.458	0.169^a^	0.087 to 0.326	83.1%^a^	67.4 to 91.3
12–23 months	12	305	7	60	0.362	0.139 to 0.944	0.766^a^	0.222 to 2.645	23.4%^a^	−100 to 77.8
**RSV-associated ICU/HDU admission**										
< 12 months	4	15	9	6	0.177	0.039 to 0.806	0.085^b^	0.004 to 1.611	91.5%^b^	−61.1 to 99.5

aOR: adjusted odds ratio; CI: confidence interval; HDU: high dependency unit; ICU: intensive care unit; Imm: immunised; OR: odds ratio; RSV: respiratory syncytial virus; Unimm: unimmunised.

^a^ Adjusted by age group, chronic medical conditions, First Nations status, grouped by week and state.

^b^ Adjusted by age group, chronic medical conditions, First Nations status, grouped by month and state.

First Nations status included all Aboriginal Australian and Torres Strait Islander people based on self-report on medical records.

## Discussion

Using an established real-time national hospital-based surveillance programme, collecting data from 22 sites in all Australian jurisdictions, we report on the 2024 RSV season. This provides the most detailed summary to date of hospitalised RSV cases in Australia across the age spectrum, including risk factors and outcomes. Moreover, the data have enabled us to assess use and effectiveness of RSV prevention products, particularly nirsevimab, which was the first to be publicly funded in Australia, and we have provided these data to policy decision makers in real-time [19].

Despite increasing testing for RSV in adult populations [20], young children remain the most likely to be admitted with RSV. In the Australian context, one in 16 children and one in 10 adults hospitalised with RSV require admission to the ICU or HDU and one in eight children and one in two adults required a hospital admission longer than 7 days. In-hospital mortality is rare in children (0.1%) but was observed in approximately 1 in 25 hospitalised adults highlighting the risk of severe outcomes for older individuals with RSV. Yet, the majority of hospitalised RSV cases require only short stays (≤ 7 days) and recover without notable complications. Our data demonstrate that children aged < 6 months, those born preterm, First Nations children and those with underlying medical conditions particularly cardiac and neurological disease, genetic or metabolic disorders, malignancy or immunosuppression are at greatest risk of severe outcomes. For adults, more severe outcomes are observed in those with malignancy, respiratory or cardiac disease. Although older adults were at lower risk of ICU/HDU admission, this is likely to be influenced by referral patterns to ICU/HDU.

We demonstrate the effectiveness of nirsevimab in jurisdictions providing population-wide programmes in 2024. Western Australia observed a > 50% reduction in hospitalised RSV cases in infants < 12 months in 2024 [21] with similar reductions observed in infants < 6 months in Queensland (personal communication, Ross Andrews, Queensland Health, April 2025). Of note, coverage estimated through surveillance of hospitalised test-negative controls recruited throughout the study period has underestimated overall population coverage. Population-wide data derived from AIR demonstrate that nirsevimab coverage exceeded 80% in newborns in both Western Australia [21] and Queensland (personal communication, Ross Andrews, April 2025). Acknowledging the risk of underestimating coverage through the assessment of test-negative controls, our data suggest that there was negligible use of both RSVpreF and RSVpreF3 OA in 2024, despite their availability on the private market. This illustrates that coverage is likely to remain low in pregnant people, older or medically-at-risk adult populations until these products are assessed and considered to be suitable for inclusion in the national immunisation programme (NIP) or jurisdictional immunisation programmes.

As of 3 February 2025, RSVpreF (Abrysvo) has been included on the NIP and recommended for every pregnant person from 28 weeks gestation [6]. Nirsevimab (Beyfortus) is recommended and funded through every Australian jurisdictional immunisation programme for neonates and infants < 8 months of age whose mothers were not vaccinated with RSVpreF (Abrysvo) at least 2 weeks prior to delivery, or infants who are at increased risk of severe disease because of maternal immunosuppression or medical risk factors (e.g. preterm birth < 32 weeks, congenital heart disease, immunosuppression, chronic lung disease requiring oxygen or respiratory support, neurological conditions impairing respiratory function, cystic fibrosis with severe lung disease or failure to thrive and genetic conditions including trisomy 21). Children 8 to 24 months with the same medical risk factors are also recommended to receive nirsevimab ahead of their second RSV season [6]. In July 2025, RSVpreF (Abrysvo) and RSVpreF3 OA (Arexvy) were recommended for all adults 75 years and older and First Nations adults 60 to 74 years by the Pharmaceutical Benefits Advisory Committee as suitable for listing on the NIP [22].

National RSV prevention programmes vary significantly by country with some favouring maternal vaccination programmes [23,24], some infant nirsevimab programmes [25-28], and a limited number of countries (such as Australia) recommending both strategies [29]. Recommendations for adult vaccination also vary. Prospective surveillance undertaken through the FluCAN-PAEDS network will be uniquely positioned to evaluate use and effectiveness of the aforementioned strategy incorporating vaccination in pregnancy and infant monoclonal antibodies and potential future use of RSV vaccines in older individual or at-risk population groups.

The strength of the network is its inclusion of 22 hospitals across the country as well as real-time, year-round data collection from all age groups. It is estimated that the surveillance network covers 20% of the national hospital bed capacity and 50% of the quaternary/tertiary bed capacity in the country [30]. This is particularly important given the variable RSV activity observed in different jurisdictions. The collection of test-negative controls is critical to enable the assessment of the effectiveness of RSV prevention products and use these data to inform current and future prevention programmes.

This study has a number of limitations. Firstly, the decision to test was left to the treating clinician using local guidelines. Although it is common practice to test for respiratory viruses in hospitalised patients with acute respiratory symptoms, and RSV is included in the majority of commercially available multiplex NAT panels, the FluCAN-PAEDS network did not influence sample collection or testing guidelines. Secondly, First Nations status and comorbidities were obtained by self report and review of the medical record respectively. Thirdly, RSV vaccines are included as a routine field on the whole-of-life Australian Immunisation Register. In Australia, immunisation data are uploaded to AIR most often though primary practice and immunisation clinic software. However, manual uploading of data is still required including from many hospital sites. Although efforts to ensure reporting of nirsevimab to the AIR were undertaken in states with RSV prevention programmes and the electronic transfer of data from primary care practice software, it is possible that immunisation status may be under-reported in a small number of RSV cases and controls. Further work is required to improve the reliability of RSV prevention product use data generated from the AIR. Lastly, the network does not collect genotyping data meaning than in-depth evaluation of immunisation breakthrough cases was not possible.

## Conclusion

In summary, we describe nearly 4,000 children and adults hospitalised with RSV in Australia in 2024, 6.7% of which required admission to ICU/HDU. RSV epidemiology varied in different jurisdictions, highly influenced by whole-of-population infant nirsevimab programmes operational in two states. Nirsevimab was highly effective at prevention RSV-hospitalisation in children < 12 months of age. The FluCAN-PAEDS network is uniquely placed to evaluate use, overall and relative effectiveness of RSV prevention products in 2025, including use of maternal RSVpreF vaccine alongside nationwide nirsevimab as part of comprehensive RSV Maternal and Infant Protection Programmes. In addition, it will provide detailed data on RSV-associated hospitalisation in older adults, informing future of RSV prevention programmes.

## Data Availability

Requests for de-identified data will be considered by the authors and shared upon reasonable request.

## Additional members of the FluCAN-PAEDS network

FluCAN: Mark Holmes, Louis Irving, Stephen Vincent, Sanjaya Senanayake, Tony Korman, Caroline Bartolo, Louise Cooley, Peter Wark, Simon Bowler, Jen Kok, Naomi Runnegar, Daniel Fatovich, Grant Waterer.

PAEDS: Anne Kynaston, Brendan McMullan, Joshua Francis, Peter Richmond.

## Supplementary Data

83000 Supplement
