# Supplementary material for: Respiratory syncytial virus epidemiology and effectiveness of infant nirsevimab: 2024 results from the Australian Sentinel Hospital Network (FluCAN-PAEDS)
Source: Euro Surveill. 2026 Jan 15;31(2):2500275. doi: 10.2807/1560-7917.ES.2026.31.2.2500275 (PMC12811708; doi:10.2807/1560-7917.ES.2026.31.2.2500275)
Supplement: Supplement [file 25-00275_BLYTH_SUPPLEMENT.pdf]

“This supplementary material is hosted by *Eurosurveillance* as supporting information alongside the article Respiratory syncytial virus epidemiology and effectiveness of infant nirsevimab: 2024 results from the Australian Sentinel Hospital Network (FluCAN-PAEDS) on behalf of the authors who remain responsible for the accuracy and appropriateness of the content. The same standards for ethics, copyright, attributions and permissions as for the article apply. *Eurosurveillance* is not responsible for the maintenance of any links or email addresses provided therein.”

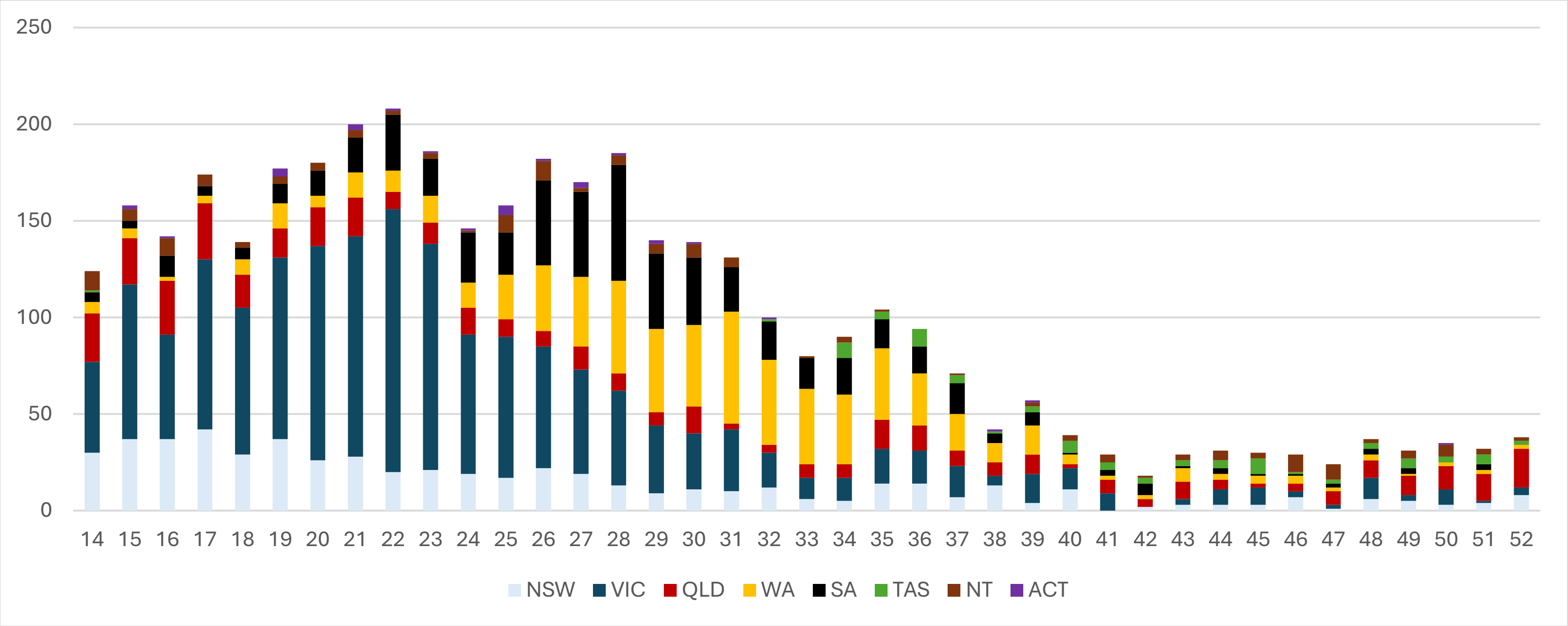

Supplemental Figure 1: Number of RSV cases per week, by jurisdiction

NSW: New South Wales; VIC: Victoria; QLD: Queensland; WA: Western Australia; SA: South Australia; TAS: Tasmania; NT: Northern Territory, ACT: Australian Capital Territory

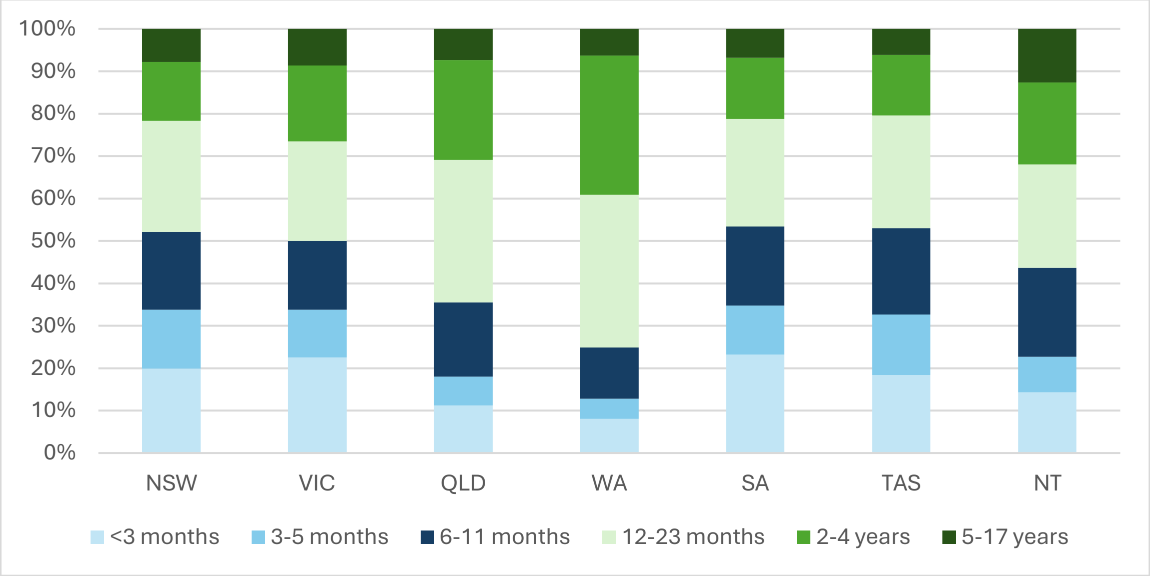

Supplemental Figure 2: Number of paediatric cases by age group and jurisdiction

NSW: New South Wales; VIC: Victoria; QLD: Queensland; WA: Western Australia; SA: South Australia; TAS: Tasmania; NT: Northern Territory
